# Neonicotinoid residues in commercial Japanese tea leaves produced by organic and conventional farming methods

**DOI:** 10.1016/j.toxrep.2021.09.002

**Published:** 2021-09-15

**Authors:** Collins Nimako, Anri Hirai, Takahiro Ichise, Osei Akoto, Shouta M.M. Nakayama, Kumiko Taira, Kazutoshi Fujioka, Mayumi Ishizuka, Yoshinori Ikenaka

**Affiliations:** a Laboratory of Toxicology, Department of Environmental Veterinary Sciences, Faculty of Veterinary Medicine, Hokkaido University, Hokkaido, Japan; b Chemistry Department, Kwame Nkrumah University of Science and Technology, Ghana; c Department of Anesthesiology, Tokyo Women’s Medical University Center East, Tokyo, Japan; d Pharmaceutical Research Institute, Albany College of Pharmacy and Health Sciences, United States; e Water Research Group, Unit for Environmental Sciences and Management, North-West University, Potchefstroom, South Africa; f One Health Research Center, Hokkaido University, Hokkaido, Japan; g Translational Research Unit, Veterinary Teaching Hospital, Faculty of Veterinary Medicine, Hokkaido University, Hokkaido, Japan

**Keywords:** NEO, neonicotinoid insecticide, JAS, Japanese Agricultural Standards, LC-ESI/MS/MS, Liquid chromatography-mass spectrometry/mass spectrometry, MRM, multiple-reaction monitoring, LOQs, Limits of quantitation, MDIgt, maximum daily intakes of NEOs via consumption of green tea leaves, LOD, limit of detection, Df, detection frequency, MAFF, Ministry of Agriculture, Forestry and Fisheries of Japan, MRLs, minimum residual levels, t_1⁄2_, half-life, ADI, acceptable daily intake, Neonicotinoid insecticide, Conventional tea leaves, Organic tea leaves

## Abstract

The current study sought to assess the residual levels of neonicotinoid insecticides (NEO) in organic and conventional green tea leaves produced in Japan. A total of 103 tea leaves (thus, 42 organic and 61 conventional), were sampled from grocery stores in Japan. Concentrations of NEOs in the tea leaves were quantified using LC–MS/MS; and the data was used to estimate maximum daily intakes of NEOs within the Japanese population. Seven native NEO compounds and one NEO metabolite were detected in both organic and conventional tea leaves. Detection frequencies (%Dfs) of NEOs in the tea samples (n = 103) were found in the decreasing order; thiacloprid (84.47 %) > dinotefuran (74.76 %) > imidacloprid (69.90 %) ≈ clothianidin (69.90 %) > dm-acetamiprid (63.11 %) > thiamethoxam (58.25 %) > acetamiprid (4.85 %) > nitenpyram (1.94 %). About 94.20 % of the tea leaves contained two or more NEO compounds simultaneously. The %Dfs of NEOs were relatively lower in organic tea leaves, compared to the conventional tea leaves. Various percentile concentrations of NEOs were far lower in organic tea leaves, compared to the conventional tea leaves. The maximum daily intakes of NEOs through consumption of tea (MDIgt) were also lower for organic tea leaves, compared to the conventional tea samples.

## Graphical abstract

**Figure:**
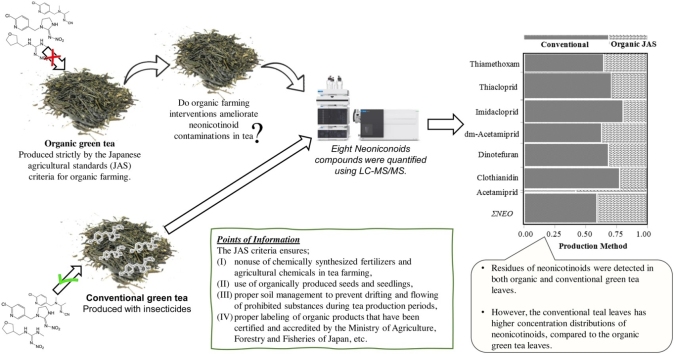

## 1 Introduction

Tea is well-known for its rich nutritional constituents such as catechins, theanine, epigallocatechin gallate, polysaccharides, vitamins, and mineral salts [1,2]. Habits of regular tea consumptions in humans, have been widely reported to result into anti-inflammatory, anticarcinogenic and antiaging effects on human health [3,4]. Moreover, a couple of scientific studies have consistently highlighted the plausible potentiation effects of tea on body weight loss, blood pressure reduction; diabetes treatment and cardiovascular disease prevention in humans [5]. As a result of these important health benefits, consumption of tea beverages has become an important component of many cultures across the world.

In Japan, tea consumption constitutes an important part of human lifestyle; especially within the older generation [6]. It has been estimated that, more than 50 % of Japanese adults consume green tea on daily basis [7]. To meet the high demand for tea in Japan, farmers often apply insecticides on tea farms in order to boost yield and to prevent post-harvest loses. However, the employment of insecticide applications in tea farming activities may result into accumulation of various residual levels of insecticides in commercial tea products for human exposure. In recent times, a plethora of scientific reports have specifically pointed out green tea consumption as a potential source of neonicotinoid insecticide (NEO) exposures within the Japanese population [[8], [9], [10], [11], [12], [13]]. Previously, Ikenaka et al. detected seven NEOs and ten NEO metabolites in commercial green tea leaves and bottled green tea beverages obtained from Japanese grocery shops [14]. The study specifically recorded high detection frequencies (%Df) of NEO metabolites such as dinotefuran-urea (92 %) and thiacloprid-amide (89 %) in the Japanese green tea samples. These staggering findings have generated serious concerns among tea consumers in Japan.

As part of regulatory responses to these concerns, the Ministry of Agriculture, Forestry and Fisheries of Japan established a national standardization system known as JAS (Japanese Agricultural Standards) [[15], [16], [17], [18], [19]]. The mandate of JAS was to, (I) elaborate of the principles of organic tea production in line with the Codex guidelines (Codex Guidelines for the Production, Processing, Labeling and Marketing of Organically Produced Foods adopted by the Codex Alimentarius Commission in 1999), and (II) label organic products that have been certified and accredited by the Ministry of Agriculture, Forestry and Fisheries of Japan [[15], [16], [17], [18], [19]]. Under the JAS system, accredited organic tea products on the Japanese market are identified by special logos (the JAS logo), embossed on their labels. Further details about the criteria adopted by JAS for organic agricultural products and their associated legislations in Japan have been presented in Table S5.

Currently, there are many tea products on the Japanese market that bear the JAS logo. The indications are that those tea products contain minimal amount of insecticidal residues. However, it is unknown whether the adoption of organic JAS farming protocols in Japan effectively attenuate insecticide contamination in tea or otherwise. Apparently, it may be highly interesting to elucidate how the organic tea production interventions in Japan affects the statues of NEO contamination reported in conventional tea products in Japan.

The objectives of the present study were (I) to determine the residual concentrations of NEOs in organic and conventional tea leaves found on the Japanese market, and (II) to evaluate the relative contamination levels of NEOs in organic tea leaves, in comparison to the NEO levels in conventional tea leaves. A total of 103 Japanese green tea leaves produced by either organic or conventional farming practices, were randomly sampled from grocery stores in Japan. Subsequently, the residual concentrations of 8 NEOs, thus; 7 native NEO compounds (acetamiprid, imidacloprid, clothianidin, dinotefuran, nitenpyram, thiamethoxam and thiacloprid) and a NEO metabolite (*N*-dm-acetamiprid [dm-acetamiprid]), were determined in the tea leave samples; and the results were compared among the tea production methods. Finally, the maximum daily exposures to the NEOs through consumption of organic or conventional tea leaves were estimated for Japanese adults and children.

## 2 Materials and methods

### 2.1 Chemicals

Clothianidin, dinotefuran, imidacloprid, acetamiprid, *N*-dm acetamiprid and thiacloprid were purchased from Kanto Chemical (Tokyo, Japan). Nitenpyram was purchased from Wako Pure Chemical Industries (Osaka, Japan). Clothianidin-d3, dinotefuran-d3, thiacloprid-d4, nitenpyram-d3, acetamiprid-d3, imidacloprid-d4, *N*-dm-acetamiprid-d3 and thiamethoxam-d4 were purchased from Sigma-Aldrich (St. Louis, MO). Thiamethoxam was purchased from Dr. Ehrenstorfer (Augsburg, Germany). All reagents and solvents used in the current study were of analytical grade; and they were purchased from Kanto Chemical Co., Inc. (Chuo-Ku, Tokyo, Japan).

### 2.2 Sampling of tea leaves

A total of 103 Japanese green tea leaves of different brands produced by different manufacturers were purchased from randomly selected grocery stores in Japan. Forty-two out of the 103 tea samples had JAS organic trademark embossed on their labels (hereafter, known as organic JAS), suggesting that these 42 tea samples were produced from organic farming systems that conformed to JAS organic criteria. The remaining 61 samples were produced from conventional farming methods, thus; farming systems that used fertilizer and synthetic insecticides on farmlands (hereafter, known as conventional). All the tea leave samples were produced in Japan between 2018 and 2021. The tea samples were stored in a refrigerator at 4 °C until analysis was performed.

### 2.3 Extraction of neonicotinoid insecticides from tea leaves

The extraction technique used in the current study was a modification of a previously published protocol [14]. Briefly, tea leaves were ground into fine powder using a mortar and pestle. A 0.1 g of the ground tea leaves was placed in a 50-mL centrifuge tube (Corning Inc., Corning, NY) and was spiked with 100 μL of NEO internal standard mix (100 ppb solution). Eight mL of distilled water was added to the sample at 25 °C. The sample was vortex-mixed thoroughly for 10–20 min; and then centrifuged at 10,000 × *g* for 10 min. After centrifugation, a 100 μL aliquot of the supernatant was carefully separated and diluted in 2900 μL of distilled water. Subsequently, the extract was loaded onto a pre-conditioned (3 mL each of acetonitrile: dichloromethane (1: 1 *v:v*) followed by 3 mL distilled water) InertSep pharma cartridge (200 mg / 3 mL, GL Sciences, Tokyo, Japan), and then washed with 0.5 mL of distilled water. Conditioning of an InertSep PSA cartridge (100 mg / 1 mL, GL Sciences, Tokyo, Japan) was then carried out with 3 mL of acetonitrile : dichloromethane (1 : 1 *v:v*) solution. The pharma and PSA cartridges were connected in series (pharma on top of the PSA cartridge); and the target analytes were eluted from the cartridges with 3 mL of acetonitrile: dichloromethane (1: 1 *v:v*) solution. After concentrating analytes with a centrifugal concentrator (CVE-200D with UT-2000; EYELA, Tokyo, Japan) at 60 °C for 1 h, the sample was redissolved in 200 μL of 3% methanol in distilled water and then transferred into vials for LC–MS/MS analysis

The tea samples were analyzed using the LC-ESI/MS/MS (Agilent 6495B, Agilent Co., CA, USA) system equipped with Kinetex Biphenyl (2.1 mm ID × 150 mm, ø 1.7 μm; Phenomenex, Inc., CA, USA). Solvents A and B used for the LC-ESI/MS/MS analysis were 0.1 % formic acid +10 mM ammonium acetate water solution and 0.1 % formic acid +10 mM ammonium acetate methanol solution, respectively. The gradient was programmed as follows: t = 0–1 min: 5% B, t = 6 min: 95 % B, t = 6–8 min: 95 % B. The column oven temperature, ﬂow rate and sample injection volume were 60 °C, 0.5 mL/min and 10 μL, respectively. The ion signals were acquired with multiple-reaction monitoring (MRM) in positive ionization mode; the selected *m/z* ions for the all the target NEO compounds considered in the current study have been shown in Table 1.

**Table 1:** Selected neonicotinoids and their metabolites.

Neonicotinoids	LOD	LOQ	Recovery rate	Precision		Linearity	MRM		CE	Polarity for
	(ng/g ww)	(ng/g ww)	±SD (%)	RSD (%)*	RSD (%)**	*r*^2^	Qualifier	Quantifier		ESI
Imidacloprid	0.07	0.20	89 ± 11.0	4	8	0.997	256.0 > 209.2	256.0 > 175.1	−15	+
Acetamiprid	0.02	0.05	101 ± 8.5	5	7	0.998	223.0 > 56.3	223.0 > 126.0	−21	+
*N*-dm- ACE	0.02	0.05	105 ± 9.6	8	6	0.998	209.1 > 72.9	209.1 > 126.1	−17	+
Clothianidin	0.03	0.10	94 ± 5.4	6	3	0.997	250.0 > 132.0	250.0 > 169.0	−16	+
Dinotefuran	0.03	0.10	96 ± 10.3	7	8	0.996	203.0 > 157.0	203.0 > 129.1	−13	+
Thiacloprid	0.44	1.33	89 ± 7.2	4	5	0.999	253.0 > 90.0	253.0 > 126.0	−15	+
Nitenpyram	0.03	0.10	95 ± 11.2	5	4	0.998	271.1 > 56.0	271.1 > 126.0	−29	+
Thiamethoxam	0.03	0.10	99 ± 6.4	5	3	0.998	292.0 > 181.0	292.0 > 211.0	−12	+

*Internal Standards*										
Clothianidin-d3	–	–	–	5	5	–	253.0 > 132.0	253.0 > 171.9	−12	+
Dinotefuran-d3	–	–	–	6	6	–	206.1 > 90.0	206.1 > 132.1	−14	+
Tiacloprid-d4	–	–	–	8	4	–	257.1 > 90.1	257.1 > 126.0	−20	+
Thiamethoxam-d4	–	–	–	4	7	–	296.1 > 185.2	296.1 > 215.1	−13	+
Nytenpyram-d3	–	–	–	6	5	–	274.1 > 126.0	274.1 > 228.2	−12	+
Acetamiprid d3	–	–	–	6	4	–	226.7 > 59.2	226.7 > 126.2	−16	+
Imidacloprid d4	–	–	–	4	8	–	260.1 > 213.1	260.1 > 179.1	−20	+
*N*-dm- ACE-d3	–	–	–	7	5	–	212.2 > 73.1	212.2 > 126.2	−24	+

*N*-dm- ACE; *N*-desmethyl-acetamiprid; LOD: limit of detection; LOQ: limit of quantification; SD: standard deviation; RSD: relative standard deviation; *intra-day precision; **inter-day precision; MRM: multiple reaction monitoring; CE: chemical equivalence; ESI: electrospray ionization.

The 8 target NEO compounds (Table 1) were analysed simultaneously in each tea sample. Results of neonicotinoid insecticides and metabolites in tea leaves were expressed in ng/g w/w.

### 2.4 Quality control and quality assurance

The tea matrix was surrogate-spiked with 8 deuterium-labelled internal standards prior to sample extraction and purification processes. The target analytes were then quantified using the internal standard method. Calibration curves were plotted from 7-point matrix-matched calibration standards, which were prepared within the concentration range of 0.05−5 ng/mL. Calibration curves were plotted using standard peak area/IS peak area ratios; and average coefficients of determination (r^2^) for the calibration curves were ≥ 0.995 (Table 1). Organic green tea leaves with undetected levels of all the target compounds were used for the preparation of matrix-matched calibration samples (Table 1). The analytical methods were checked for precision and accuracy. Eight NEO compounds were detected with recovery rates ranging from 89 to 105 %, as shown in Table 1. Precision of the analytical technique was confirmed by inter-day and intra-day analysis; and the relative standard deviations recorded in each case were less than 10 % for all the target compounds (Table 1). Limits of detection (LOD) and limits of quantitation (LOQ) were calculated as the lowest points on the standard curves (Table 1) with relative standard deviations of less than 10 % (*n* = 5) and signal-to-noise ratios of 3:1 and 9:1, respectively.

### 2.5 Maximum daily intake of neonicotinoids through green tea leaves

In the present study, the maximum daily intakes of NEOs via consumption of green tea leaves (MDIgt) were estimated using the relation below [14].

$$MDIgt=\;\frac{Cmax\;\times \;LP}{Bw}$$

Where Cmax represents the maximum concentration of the target NEOs detected in tea leaves (mg/g w/w); LP represents the 97.5th percentile of daily consumption of tea leaves estimated for Japanese adults and children (1–6 years old), 35.7 g and 15.3 g were assumed, respectively [14]; and Bw is the average body weight (kg), 57.0 kg (adults) and 16.2 kg (children) were assumed [14]; in this study.

### 2.6 Statistical analysis

Data analysis was performed using JMP Pro14 (SAS Institute Inc., Cary, NC, USA). The data were tested for normality using the Shapiro-Wilk test; and for homogeneity of variance using the Levene’s test. During the estimations of means, percentile concentrations and *%Df,* concentrations of NEOs below LOD were set to zero. When testing for statistical significance however, concentrations of NEOs below LOD were assigned to the LOD values (Table 1) divided by square root of 2 [20]. Significant differences between median concentrations of NEOs were tested using the nonparametric 1-Way Median Test, with Chi-square approximation, *p* <0.0001. Spearman Rho`s nonparametric correlation test was also employed to determine the correlations between concentrations of NEOs.

## 3 Results and discussion

### 3.1 Neonicotinoid concentrations in the overall tea leave samples (n = 103)

In the present study, 8 NEOs (acetamiprid, imidacloprid, dinotefuran, clothianidin, thiamethoxam, nitenpyram, thiacloprid and dm-acetamiprid) were detected in Japanese green tea leaves (Table 2). The detection frequencies of NEOs in the overall tea leave samples (n = 103) were found in the decreasing order; thiacloprid (84.47 %) > dinotefuran (74.76 %) > imidacloprid (69.90 %) ≈ clothianidin (69.90 %) > dm-acetamiprid (63.11 %) > thiamethoxam (58.25 %) > acetamiprid (4.85 %) > nitenpyram (1.94 %) *(*Table 2).

**Table 2:** Concentrations of neonicotinoids detected in tea leaves.

Neonicotinoid	Production		Percentile concentrations (ng/g ww)				
	method	%Df	25th	50th	75th	95th	100th
Acetamiprid	Organic JAS (n = 41)	4.88	BDL	BDL	BDL	0.36	1.27
	Conventional (n = 62)	4.84	BDL	BDL	BDL	BDL	14.41
	Overall (n = 103)	4.85	BDL	BDL	BDL	0.11	14.41
dm-Acetamiprid	Organic JAS (n = 41)	56.10	BDL	0.35	0.77	1.24	1.27
	Conventional (n = 62)	67.74	BDL	0.40	0.52	0.74	0.78
	Overall (n = 103)	63.11	BDL	0.36	0.54	1.03	1.27
Clothianidin	Organic JAS (n = 41)	36.59	BDL	BDL	0.95	3.33	6.30
	Conventional (n = 62)	91.94	2.02	6.92**	19.69	127.53	328.59
	Overall (n = 103)	69.90	BDL	2.02	10.49	83.81	328.59
Dinotefuran	Organic JAS (n = 41)	58.54	BDL	0.69	3.39	10.99	27.29
	Conventional (n = 62)	85.48	9.31	59.64**	246.67	1113.38	3407.24
	Overall (n = 103)	74.76	0.10	8.30	108.13	836.77	3407.24
Imidacloprid	Organic JAS (n = 41)	34.15	BDL	BDL	0.90	4.94	6.70
	Conventional (n = 62)	93.55	1.22	2.30**	3.17	5.57	14.16
	Overall (n = 103)	69.90	BDL	1.45	2.75	5.54	14.16
Nitenpyram	Organic JAS (n = 41)	4.88	BDL	BDL	BDL	BDL	0.08
	Conventional (n = 62)	BDL	BDL	BDL	BDL	BDL	0.01
	Overall (n = 103)	1.94	BDL	BDL	BDL	BDL	0.08
Thiacloprid	Organic JAS (n = 41)	60.98	BDL	0.30	0.38	1.11	3.48
	Conventional (n = 62)	100.00	0.22	0.30	0.41	2.29	11.99
	Overall (n = 103)	84.47	0.22	0.30	0.39	1.87	11.99
Thiamethoxam	Organic JAS (n = 41)	51.22	BDL	0.21**	0.88	3.88	126.18
	Conventional (n = 62)	62.90	BDL	1.25	10.44	211.08	959.40
	Overall (n = 103)	58.25	BDL	0.63	3.61	101.46	959.40
*ΣNEO*	Organic JAS (n = 41)	–	1.26	3.21**	8.41	29.30	150.54
	Conventional (n = 62)	–	20.96	148.50	373.29	1222.04	3449.09
	Overall (n = 103)	–	3.35	17.63	187.48	1127.53	3449.09

*BDL means below the detection limit; %DF: percentage detection frequency; ΣNEO: total neonicotinoid concentration. Significant difference between the organic and conventional tea leaves, nonparametric Median Test, 1-Way Test, Chi-square approximation, ** *p* <0.0001. Data < LOD were not considered while calculating %Df, means and percentile concentrations. During statistical significance analysis concentrations of NEOs below LOD were set as the LOD value divided by the square root of 2 [20].

The highest 50th percentile concentration of NEOs was recorded for dinotefuran (8.30 ng/g w/w), followed by clothianidin (2.02 ng/g w/w), imidacloprid (1.45 ng/g w/w), thiamethoxam (0.63 ng/g w/w), dm-acetamiprid (0.36 ng/g w/w) and then, thiacloprid (0.30 ng/g w/w) (Table 2). However, the 50th percentile concentrations of acetamiprid and nitenpyram were found below detection limit. The maximum concentrations of NEOs detected in the tea leaves were found in the decreasing order; dinotefuran (3407.24 ng/g w/w) > thiamethoxam (959.40 ng/g w/w) > clothianidin (328.59 ng/g w/w) > acetamiprid (14.41 ng/g w/w) > imidacloprid (14.16 ng/g w/w) > thiacloprid (11.99 ng/g w/w) > dm-acetamiprid (1.27 ng/g w/w) > nitenpyram (0.08 ng/g w/w) (Table 2). These results suggest that the use of NEO containing formulations, especially, thiacloprid, dinotefuran, imidacloprid and clothianidin, are quite prevalent in most tea farming systems in Japan.

Previously, imidacloprid was considered as most patronised NEO in the world. In recent times however, imidacloprid has been replaced with thiamethoxam and clothianidin in many agricultural systems across the world [21]. In Japan, dinotefuran was considered as the most consumed NEO in 2010, followed by acetamiprid, clothianidin, imidacloprid and thiamethoxam [12]. Hence the detection trends of dinotefuran, clothianidin and thiamethoxam observed in the current study, fairly reflects the domestic consumption patterns of NEOs in Japan.

The predominance of dinotefuran detection trends observed in the current study agrees with findings from a previous study [14], which similarly recorded the highest maximum concentrations for dinotefuran (Df = 100 %, 3004 ng/g w/w), among various NEO compounds measured in Japanese green tea leaves. Ikenaka et al.*’s* group further detected high frequencies and high maximum concentrations for dinotefuran metabolites such as dinotefuran-urea (Df = 87 %, 77.1 ng/g w/w) and *N*-dm-dinotefuran (Df = 10 %, 9.4 ng/g w/w) [14]. These consistent observations suggest that most conventional tea farming systems in Japan probably employ the use of dinotefuran containing formulations on frequent basis. The Ministry of Agriculture, Forestry and Fisheries of Japan (MAFF) permits the applications of NEOs such as dinotefuran, clothianidin and thiamethoxam on tea farms from the unset of cultivation until seven days before harvest [22]. Among these 3 NEOs however, only dinotefuran can be applied 2 times within the pre-harvest period. These provisions probably explain the predominance of dinotefuran levels observed in the current study.

About 94.20 % of the tea leaves used in the current study, were found to contain two or more NEO compounds simultaneously (Fig. 2A). This suggests that majority of tea farming systems in Japan apply multiple NEO containing formulations at a time. It is noteworthy that, the maximum concentrations of all the NEOs detected in the current study were found below their respective minimum residual levels (MRLs, Tables 2 and S3). However, this does not entirely guarantee an absence of toxicological implications of NEOs on human health via tea consumption. This is because toxicological risk assessment in food depends on both (I) concentration of the pollutant in food; and (II) consumption rates [14,23].

Supplementary Data, Table S6 shows comparisons between concentrations of 8 NEOs detected in the overall green tea leave samples (current study, n = 103) and the urinary concentrations of the target NEOs in Japanese and/or other human populations across the world. From the data, dinotefuran had dominant detection rates both in green tea leaves and in Japanese urine [[24], [25], [26]]. This strongly confirms that dinotefuran dominates in the domestic applications of NEOs in Japan. The median concentration of all the target NEOs recorded in the green tea leaves were higher than the urinary NEO concentrations reported in Japanese [[24], [25], [26]] and other populations in Ghana [27], China [28,29] and USA [30]. Apart from imidacloprid and nitenpyram, the maximum concentrations of all the NEO parent compounds (acetamiprid, clothianidin, dinotefuran, thiacloprid and thiamethoxam) detected in the current green tea samples were higher than their respective urinary concentrations reported in urine of Japanese, Ghanaians, Chinese and Americans [[24], [25], [26], [27], [28], [29], [30]], as shown in Supplementary Data, *Table S6.* Moreover, the total concentrations of NEOs recorded in the green tea leaves were far higher than the cumulative urinary levels of NEOs reported in the various human populations (Supplementary Data, Table S6, [[24], [25], [26], [27], [28], [29], [30]]). Urinary levels of NEOs were lower in human urine compared to NEO residues in tea leaves probably because of enzyme-mediated metabolism and subsequent excretions of NEOs in humans. Moreover, most of the reported human studies quantified NEOs in spot urine samples rather than 24 -h urine samples. Spot urinary contaminant data alone are of uncertain value because of highly variable dilutions that may be instigated by wide fluctuations of fluid intakes [31]. Hence, the reported urinary concentrations observed in the human subjects [[24], [25], [26], [27], [28], [29], [30]] may not entirely reflect the total urinary contents of NEOs in the various human populations. Meanwhile, the maximum urinary concentration of dm-acetamiprid reported in Japanese (53.3 ng/mL [25]), Ghanaians (8.79 ng/mL [27]) and Chinese (18.3 ng/mL [29]), were found to be far higher than the maximum dm-acetamiprid concentration recorded in the green tea leaves (1.27 ng/g) used for the current study (Supplementary Data, *Table S6*). This tendency was probably because of the prolific metabolic formation of dm-acetamiprid from acetamiprid in humans by CYP450 enzymes [32].

### 3.2 Impacts of farming methods on neonicotinoid levels in tea leaves

**Fig. 1:**
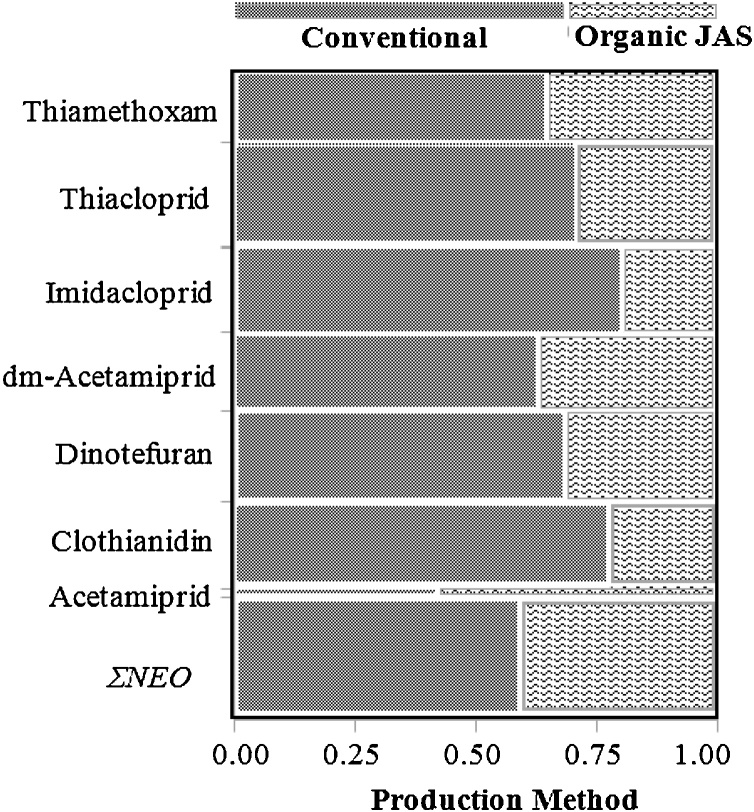
Distribution of mean concentrations of neonicotinoids in tea leaves.

Impacts of organic and conventional tea farming methods on residual levels of NEOs in Japanese tea leaves were evaluated by comparing the detection frequencies and distribution frequencies of NEOs among the two farming methods *(*Table 2 and Fig. 1). Interestingly, all the target NEOs were detected with appreciable frequencies in both organic and conventional tea leaves (Table 2). Particularly, significant detections of thiacloprid, dinotefuran, dm-acetamiprid, thiamethoxam, clothianidin and imidacloprid were observed in the organic JAS tea leaves (*Dfs* of 60.98 %, 58.54 %, 56.10 %, 51.22 %, 36.59 % and 34.15 % respectively). This finding was considered puzzling in that, the JAS criteria for organic tea farming involves strict compliance with stringent measures and/ legislations that are well-instituted to reduce insecticidal residues in food to negligible levels (Table S5). Plausibly, the observed prevalence rates of NEOs in organic tea leaves were because of contaminations from soils, interstitial/ground water, or as a result of contamination from surface water used form irrigational activities. Previous studies have highlighted NEO contaminations in agricultural soils and urban soils [[31], [32], [33]]. Other studies have also reported soil erosion-related transport of NEOs in agricultural fields; and NEO contaminations in waters/sediments systems [34,35]. Although the JAS organic tea farming criteria requires a 2–3-year fallow period for pre-cultivated soils [36], such periods may not necessarily guarantee absolute depletions of chemicals which have long half-lives in soils. Neonicotinoids compounds have varying half-lives (t_1⁄2_) in soils; with clothianidin having the longest (13–1386 days). This is followed by imidacloprid (t_1⁄2_ = 104–228 days), dinotefuran (t_1⁄2_ = 82 days), thiamethoxam (t_1⁄2_ = 7–72 days) and thiacloprid (t_1⁄2_ = 9–27 days) (Table S4, [27,37]. Acetamiprid has the shortest soil half-life (t_1⁄2_ = 4–7 days); perhaps, this explains its low detection rates in the tea leaves.

**Fig. 2:**
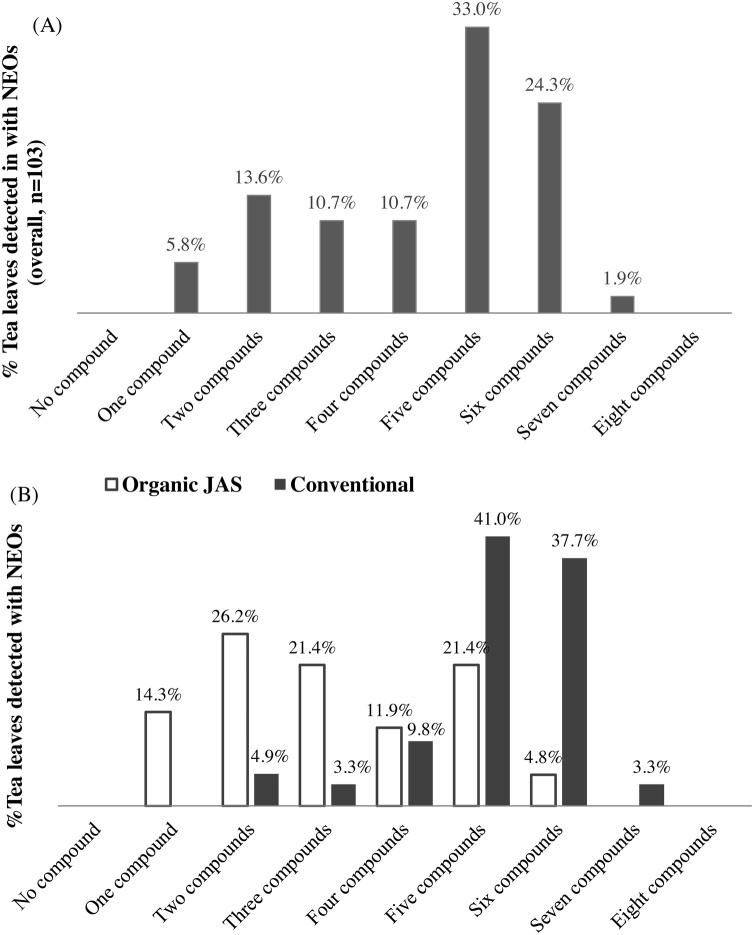
A&B: Multiple neonicotinoid detections in (A) the overall green tea samples (n = 103), (B) organic verses conventional tea leaves (For interpretation of the references to colour in this figure legend, the reader is referred to the web version of this article).

The detection frequencies (%Dfs) of dinotefuran, clothianidin, imidacloprid, thiamethoxam, dm-acetamiprid and thiacloprid (which were the most predominantly detected NEOs in the current study) were relatively lower in organic tea leaves, compared to their respective %Dfs in the conventional tea leaves *(*Table 2*)*. Also, mean concentrations of the detected NEOs were more distributed in the conventional tea leaves, than the organic tea leaves (Fig. 1). In confirmation to this observation, various percentile concentrations of NEOs such as dinotefuran, clothianidin, imidacloprid, thiamethoxam and thiacloprid recorded in conventional tea leaves were found to be far higher than those obtained in organic tea leaves *(*Table 2*)*. The differences in median concentrations of NEOs between the organic and conventional tea leaves were statistically significant (nonparametric Median Test, 1-Way Test, Chi-square approximation, *p* < 0.0001, Table 2). These observations clearly suggest that the adoption and implementation of organic farming practices in Japanese tea production resulted into drastic reductions in residual neonicotinoid concentrations in commercial green tea leaves.

Primarily, the growth conditions of the tea plant (*Camellia sinensis L*) are known to be favourable for pests, diseases, and competing grasses. As such, conventional tea farming practices often involve the applications of fertilizers and assorted insecticides on farmlands, as a measure to prevent pests and disease; and to improve tea yield and quality [1,5,38]. This may, however, culminate into increased residual levels of pesticides in tea leaves. Ikenaka et al. detected seven (7) neonicotinoid insecticides and ten (10) neonicotinoid metabolites in in Japanese tea leaves and Japanese bottled green tea beverages [14]. Another study by Chen et al. [1] detected acetamiprid, imidacloprid, buprofezin, carbedazim, and pyredaben in tea leaves on Chinese market. Hayward et al. also detected various pesticides within the concentration range of 1–3200 μg/kg in commercial tea products in US [4]. These findings are strongly confirmatory that, tea leaves produced from different part of the globe, could store high residual levels of various pesticides for human exposure.

Adoption of organic farming system is a well-trumpeted counter measure to pesticide exposures through food. In Japan, the green farming methods (organic farming methods) have been well standardized and promoted nationwide, through the JAS system. In the current study, the organic Japanese tea samples showed attenuated NEO residues, compared to the conventional tea leaves (Fig. 2B). Whereas 95.1 % of the conventional tea samples contained > 3 NEO residues at same time, only 59.5 % of the organic tea samples were found to contain > 3 NEO compounds (Fig. 2B). Moreover, about 82 % of the conventional tea samples were found to contain 5–7 NEO compounds, but only 26.2 % of the organic tea samples contained 5–7 NEO compounds simultaneously (Fig. 2B). These strongly confirm that the adoption of organic farming systems in Japan might have efficiently reduced the occurrence of residual NEO infiltrations into Japanese tea products.

After statistical analysis, only four NEO pairs exhibited moderate to strong positive correlations in organic tea leaves (thiamethoxam/acetamiprid: *r* = 0.380, *p* < 0.05; thiamethoxam/dinotefuran: *r* = 0.493, *p* > 0.05; dinotefuran/clothianidin: *r* = 0.530, *p* < 0.001; nitenpyram/acetamiprid: *r* = 0.651, *p* < 0.001), as shown in Supplementary Data, Table S1. On the other hand, about nine pairs of NEOs showed moderate to very strong correlations with each other in the conventional tea leaves (dinotefuran/clothianidin: *r* = 0.293, *p* < 0.05; imidacloprid/dinotefuran: *r* = 0.325, *p* < 0.05; thiacloprid/imidacloprid: *r* = 0.341, *p* < 0.05; thiamethoxam/imidacloprid: *r* = 0.354, *p* < 0.05; thiacloprid/dinotefuran: *r* = 0.356, *p* < 0.05; thiamethoxam/clothianidin: *r* = 0.383, *p* < 0.05; thiamethoxam/thiacloprid: *r* = 0.413, *p* < 0.001; thiamethoxam/dinotefuran: *r* = 0.451, *p* < 0.001; nitenpyram/acetamiprid: *r* = 0.841, *p* < 0.001), as shown in Supplementary Data, Table S2. These observations suggest that sources of NEO residues in conventional tea leaves are more common and consistent, compared to NEO sources in the organic tea leaves.

### 3.3 Estimation of maximum dietary intake of neonicotinoid insecticides through green tea leaves

In the present study, MDIgts of NEOs were estimated for tea samples cultivated by various farming methods in Japan; and the results have been shown in Table 3. Subsequently, the MDIgts of NEOs obtained in the present study were compared with the current Acceptable Daily Intakes (ADI) of NEOs recommended by the Food Safety Commission of Japan (Table 3 [14]).

**Table 3:** Daily intakes (μg/kg/day) of neonicotinoid through consumption of Japanese tea leaves.

Neonicotinoid	Production	ADI	MDIgt_Adult		MDIgt_Child	
	method	μg/kg/day	μg/kg/day	ADI%	μg/kg/day	ADI%
*ΣACE*	Organic JAS (n = 41)		1.59E-03	0.00	2.39E-03	0.00
	Conventional (n = 62)	71	9.52E-03	0.01	1.44E-02	0.02
	Overall (n = 103)		9.82E-03	0.01	1.48E-02	0.02
Clothianidin	Organic JAS (n = 41)		3.95E-03	0.00	5.95E-03	0.01
	Conventional (n = 62)	97	2.06E-01	0.21	3.10E-01	0.32
	Overall (n = 103)		2.06E-01	0.21	3.10E-01	0.32
Dinotefuran	Organic JAS (n = 41)		1.71E-02	0.01	2.58E-02	0.01
	Conventional (n = 62)	220	2.13E+00	0.97	3.22E+00	1.46
	Overall (n = 103)		2.13E+00	0.97	3.22E+00	1.46
Imidacloprid	Organic JAS (n = 41)		4.20E-03	0.01	6.33E-03	0.01
	Conventional (n = 62)	57	8.87E-03	0.02	1.34E-02	0.02
	Overall (n = 103)		8.87E-03	0.02	1.34E-02	0.02
Nitenpyram	Organic JAS (n = 41)		5.03E-05	0.00	7.59E-05	0.00
	Conventional (n = 62)	530	3.53E-06	0.00	5.33E-06	0.00
	Overall (n = 103)		5.03E-05	0.00	7.59E-05	0.00
Thiacloprid	Organic JAS (n = 41)		2.18E-03	0.02	3.28E-03	0.03
	Conventional (n = 62)	12	7.51E-03	0.06	1.13E-02	0.09
	Overall (n = 103)		7.51E-03	0.06	1.13E-02	0.09
Thiamethoxam	Organic JAS (n = 41)		7.90E-02	0.44	1.19E-01	0.66
	Conventional (n = 62)	18	6.01E-01	3.34	9.06E-01	5.03
	Overall (n = 103)		6.01E-01	3.34	9.06E-01	5.03
*ΣMDIgt*	Organic JAS (n = 41)		1.08E-01	–	1.63E-01	–
	Conventional (n = 62)	–	2.97E+00	–	4.47E+00	–
	Overall (n = 103)		2.97E+00	–	4.47E+00	–

MDIgt means maximum daily intakes of the target NEOs via consumption of green tea leaves; MDIgt was estimated from the 100th percentile concentrations of neonicotinoids in tea leaves; *ΣACE* sum of concentrations of acetamiprid and dm-acetamiprid in green tea leaves.

The MDIgt of NEOs obtained from the organic farming methods were generally lower, compared to those obtained from the conventional farming practices (Table 3). This indicates that human exposures to NEOs through consumption of organic tea leaves in Japan are minimal, compared to NEO exposures through conventional tea leaves. Also, MDIgt percent of ADI (%ADI) of NEOs obtained from the organic tea leaves were lower, compared to those obtained from the conventional tea samples. This suggest that the plausibly toxicological risks posed by NEOs to humans through organic tea consumptions are negligible, compared to the risks associated with the consumption of conventional tea leaves.

The %ADIs of NEOs estimated in adults and children were highest for thiamethoxam (3.34 %, 5.03 %, respectively) followed by dinotefuran (0.97, 1.46 %, respectively), clothianidin (0.21, 0.32, respectively), thiacloprid (0.06 %, 0.09 %, respectively), imidacloprid (0.02 %, 0.02 %, respectively), acetamiprid (0.01 %, 0.02 %, respectively) and nitenpyram (0.00 %, 0.00, respectively) (Table 3). However, the cumulative daily exposures to NEOs (ΣMDIgts) estimated for children were relatively higher, compared to those obtained for adults. Moreover, the %ADIs of all the target NEOs were comparatively higher in children than in adults. Ultimately, these results suggest that the potential risks of NEOs posed to children through daily consumption of tea products are greater compared to that of adults. This finding agreed with a previous report which similarly found higher NEO risks to children, as a result to green tea consumption [14]. Children may highly be susceptible to adverse health effects of NEOs than adults, due to their higher dietary consumption rates than adults.

## 4 Conclusions

Eight NEO compounds (clothianidin, dinotefuran, thiacloprid, imidacloprid, acetamiprid, nitenpyram, thiamethoxam and dm-acetamiprid) were detected in both JAS labelled organic tea leaves and conventional tea leaves from Japan. The NEOs were more frequently detected in conventional tea leaves than the organic tea leaves. Concentrations of NEOs detected in conventional tea leaves were extremely high, compared to their respective levels in organic tea leaves. The current study further revealed high daily exposure rates of NEOs among Japanese children and adults who patronize conventional tea leaves than those who consume organic tea leaves. Meanwhile, children were found to be more susceptible to maximum daily exposures of NEOs within the Japanese population.

Tea is a global beverage consumed in many human populations across the world. Hence, the occurrence of pesticide contaminations in tea leaves trigger serious concerns from many parts of the world. The present study presents robust scientific evidence that may be essential for validating the effectiveness of organic farming methods as a counter measure to pesticide contaminations in tea leaves.

## Data accessibility

Data, associated metadata, and calculation tools are available by contacting the corresponding author (y_ikenaka@vetmed.hokudai.ac.jp).

## CRediT authorship contribution statement

**Collins Nimako:** Conceptualization, Investigation, Methodology, Writing - original draft. **Anri Hirai:** Validation, Investigation. **Takahiro Ichise:** Validation, Investigation. **Osei Akoto:** Supervision. **Shouta M.M. Nakayama:** Conceptualization, Supervision. **Kumiko Taira:** Funding acquisition, Writing - review & editing. **Kazutoshi Fujioka:** Writing - review & editing. **Mayumi Ishizuka:** Conceptualization, Funding acquisition, Supervision. **Yoshinori Ikenaka:** Conceptualization, Investigation, Project administration, Supervision.

## Declaration of Competing Interest

The authors declare that they have no known competing financial interests or personal relationships that could have appeared to influence the work reported in this paper.

## References

[1] Chen H., Gao G., Chai Y., Ma G., Hao Z., Wang C., Liu X., Lu C. (2017). Multiresidue method for the rapid determination of pesticide residues in tea using ultra performance liquid chromatography orbitrap high resolution mass spectrometry and in-syringe dispersive solid phase extraction. ACS Omega.

[2] Wada K., Oba S., Tsuji M., Goto Y., Mizuta F., Koda S., Uji T., Hori A., Tanabashi S., Matsushita S., Tokimitsu N., Nagata C. (2019). Green tea intake and colorectal cancer risk in Japan: the Takayama study. Jpn. J. Clin. Oncol..

[3] Cajka T., Bachanova V., Drabova L., Kalachova K., Pulkrabova J., Hajslova J. (2012).

[4] Hayward G.D., Wong W.J., Park Y.J. (2015). Determinations for pesticides on black, green, oolong, and white teas by gas chromatography triple-quadrupole mass spectrometry. J Agr Food Chem..

[5] Ly T., Ho T., Behra P., Nhu-Trang T. (2020). Determination of 400 pesticide residues in green tea leaves by UPLC-MS/MS and GC-MS/MS combined with QuEChERS extraction and mixed-mode SPE clean-up method. Food Chem..

[6] Abe S.K., Saito E., Sawada N., Tsugane S., Ito H., Lin Y., Tamakoshi A., Sado J., Katamura Y., Sagawara Y., Tsuji I., Nagata C., Sadakane A., Shimazu T., Mizoue T., Matsuo K., Naito M., Tanaka K., Inoue M. (2019). Green tea consumption and mortality in Japanese men and women: a pooled analysis of eight population-based cohort studies in Japan. Eur. J. Epidemiol..

[7] Wang L., Gong L.-H., Chen C.-J., Han H.-B., Li H.-H. (2012). Column-chromatographic extraction and separation of polyphenols, caﬀ ;eine and theanine from green tea. Food Chem..

[8] Marfo J.T., Fujioka K., Ikenaka Y., Nakayama S.M., Mizukawa H., Aoyama Y., Ishizuka M., Taira K. (2015). Relationship between urinary N-desmethyl-acetamiprid and typical symptoms including neurological ﬁndings: a prevalence case-control study. PLoS One.

[9] Taira K., Aoyama Y., Kawakami T., Kamata M., Aoi T. (2011). Detection of chloropyridinyl neonicotinoid insecticide metabolite 6-chloronicotinic acid in the urine: six cases with subacute nicotinic symptoms. Jpn. J. Toxicol..

[10] Taira K. (2012). Health eﬀ ;ects of neonicotinoid insecticides-Part1: physicochemical characteristics and case reports. Jpn. J. Clin. Ecol..

[11] Taira K., Fujioka K., Aoyama Y. (2013). Qualitative proﬁling and quantiﬁcation of neonicotinoid metabolites in human urine by liquid chromatography coupled with mass spectrometry. PLoS One.

[12] Taira K. (2014). Human neonicotinoids exposure in Japan. Jpn. J. Clin. Ecol..

[13] Nakanishi R., Hamada S., Ohfuji M., Owaki S., Kobayashi S., Higuchi Y. (2013).

[14] Ikenaka Y., Fujioka K., Kawakami T., Ichise I., Bortey-Sam N., Nakayama S.M.M., Mizukawa H., Taira K., Takahashi K., Kato K., Arizono K., Ishizuka K. (2018). Contamination by neonicotinoid insecticides and their metabolites in Sri Lankan black tea leaves and Japanese green tea leaves. Toxicol. Rep..

[15] (2005).

[16] Ministry of Agriculture, Forestry and Fisheries (2005).

[17] Ministry of Agriculture, Forestry and Fisheries (2007). https://www.maff.go.jp/e/policies/standard/specific/organic_JAS.html.

[18] (2007).

[19] Ministry of Agriculture, Forestry and Fisheries. Japanese Agricultural Standards (JAS). Available online: http://www.maff.go.jp/soshiki/syokuhin/hinshitu/e_label/specificJAS-organic.htm. (Accessed on 08 January 2021).

[20] Hornung R.W., Reed L.D. (1990). Estimation of average concentration in the presence of nondetectable values. Appl. Occup. Environ. Hyg..

[21] Simon-Delso N., Amaral-Rogers V., Belzunces L.P., Bonmatin J.M., Chagnon M., Downs C., Furlan L., Gibbons D.W., Giorio C., Girolami V., Goulson D., Kreutzweiser D., Krupke C.H., Liess M., Long E., McField M., Mineau P., Mitchell E.A., Morrissey C.A., Noome D.A., Pisa L., Settele J., Stark D.J., Tapparo A., Van Dyck H., Van Praagh J., Van der Sluijs P.J., Whitehorn R.P., Wiemers M. (2015). Systemic insecticides (neonicotinoids and fipronil): trends, uses, mode of action and metabolites. Environ. Sci. Pollut. Res. Int..

[22] (2016).

[23] Wongsasuluk P., Chotpantarat S., Siriwong W., Robson M. (2014). Heavy metal contamination and human health risk assessment in drinking water from shallow groundwater wells in an agricultural area in Ubon Ratchathani province, Thailand. Environ. Geochem. Health.

[24] J. Ueyama, H.K. Harada, A. Koizumi, Y. Sugiura, T. Kondo, I. Saito, M. Kamijima, Temporal level of urinary neonicotinoid and dialkylphosphate concentrations in Japanese women between 1994 and 2011. Environ. Sci. Technol. 49(201, 14522–14528. 10.1021/acs.est.5b03062 26556224

[25] Osaka A., Ueyama J., Kondo T., Nomura H., Sugiura Y., Saito I., Nakane K., Takaishi A., Ogi H., Wakusawa S., Ito Y., Kamijima M. (2016). Exposure characterization of three major insecticide lines in urine of young children inJapan-neonicotinoids, organophosphates, and pyrethroids. Environ. Res..

[26] Oya N., Ito Y., Ebara T., Kato S., Ueyama J., Aoi A., Nomasa K., Sato H., Matsuki T., Sugiura-Ogasawara M., Saitoh S., Kamijima M. (2021). Cumulative exposure assessment of neonicotinoids and investigation into their intake-related factors in young children in Japan. Sci. total Environ..

[27] Nimako C., Ikenaka Y., Akoto O., Bortey-Sam N., Ichise T., Nakayama S.M.M., Asante A.K., Fujioka K., Taira K., Ishizuka M. (2021). Human exposures to neonicotinoids in Kumasi. Ghana. Environ Toxicol Chem..

[28] Zhang T., Song S., Bai X., He Y., Zhang B., Gui M., Kannan K., Lu S., Huang Y., Sun H. (2019). A nationwide survey of urinary concentrations of neonicotinoid insecticides in China. Environ. Int..

[29] Wang A., Mahai G., Wan Y., Yang Z., He Z., Xu S., Xia W. (2020). Assessment of imidacloprid related exposure using imidacloprid-oleﬁn and desnitro-imidacloprid: neonicotinoid insecticides in human urine in Wuhan. China. Environ. Int..

[30] Honda M., Robinson R., Kannan K. (2019). A simple method for the analysis of neonicotinoids and their metabolites in human urine. Environ. Chem..

[31] Mage D.T., Allen R.H., Anuradha K. (2008). Creatinine corrections for estimating children’s and adult’s pesticide intake doses in equilibrium with urinary pesticide and creatinine concentrations. J. Expo. Sci. Environ. Epidemiol..

[32] Harada H.K., Tanaka K., Sakamoto H., Imanaka M., Niisoe T., Hitomi T., Kobayashi H., Okuda H., Inoue S., Kusakawa K., Oshima M., Watanabe K., Yasojima M., Takasuga T., Koizumi A. (2016). Biological monitoring of human exposure to neonicotinoids using urine samples, and neonicotinoid excretion kinetics. PLoS One.

[33] Humann‐Guilleminot S., Binkowski Ł., Jenni L., Hilke G., Glauser G., Helfenstein F. (2019). A nation‐wide survey of neonicotinoid insecticides in agricultural land with implications for agri‐environment schemes. J. Appl. Ecol..

[34] Zhang P., Ren C., Sun H., Min L. (2018). Sorption, desorption and degradation of neonicotinoids in four agricultural soils and their effects on soil microorganisms. Sci. Total Environ..

[35] Niu Y.H., Li X., Wang H.X., Liu Y.J., Shi Z.H., Wang L. (2020). Soil erosion-related transport of neonicotinoids in new citrus orchards. Agric. Ecosyst. Environ..

[36] Organic Soil (OS). About JAS Organic Agricultural Products. Available online: http://www.shizennoho.co.jp/business/jas_yuuki.html (accessed on 10 May 2021).

[37] Raina-Fulton R. (2016). https://www.researchgate.net/profile/Renata_Raina-Fulton_bailey/publication/309174307_Neonicotinoid_Insecticides_Environmental_Occurrence_in_Soil_Water_and_Atmospheric_Particles/links/580248e008ae6c2449f7f937.

[38] Amirahmadi M., Shoeibi S., Abdollahi M., Rastegar H., Khosrokhavar R., Hamedani M.P. (2013). Monitoring of some pesticide in consumed tea in Tehran market. Iranian J. Environ. Health Sci. Eng..

