# Supplementary material for: Neonicotinoid residues in commercial Japanese tea leaves produced by organic and conventional farming methods
Source: Toxicol Rep. 2021 Sep 15;8:1657–64. doi: 10.1016/j.toxrep.2021.09.002 (PMC8456056; doi:10.1016/j.toxrep.2021.09.002)
Supplement: Supplementary file 1 [file mmc1.docx]

**Supplementary Data**

**Table S1:** Spearman rho`s correlation analysis of neonicotinoid concentrations in organic green tea leaves (n=42)

|  | Acetamiprid | Clothianidin | Dinotefuran | dm-acetamiprid | Imidacloprid | Nitenpyram | Thiacloprid | Thiamethoxam |
| --- | --- | --- | --- | --- | --- | --- | --- | --- |
| Acetamiprid | 1.000 |  |  |  |  |  |  |  |
| Clothianidin | 0.070 | 1.000 |  |  |  |  |  |  |
| Dinotefuran | 0.018 | 0.530** | 1.000 |  |  |  |  |  |
| dm-acetamiprid | -0.257 | -0.257 | -0.544** | 1.000 |  |  |  |  |
| Imidacloprid | 0.198 | 0.281 | 0.298 | -0.249 | 1.000 |  |  |  |
| Nitenpyram | 0.651** | -0.003 | 0.137 | 0.175 | 0.176 | 1.000 |  |  |
| Thiacloprid | -0.191 | -0.076 | 0.105 | -0.213 | 0.302 | -0.060 | 1.000 |  |
| Thiamethoxam | 0.060 | 0.380* | 0.493* | -0.341 | 0.100 | -0.020 | -0.223 | 1.000 |

Spearman Rho`s nonparametric correlation test; ***** *p* > 0.05, ***p* > 0.001

**Table S2:** Spearman rho`s correlation analysis of neonicotinoid concentrations in conventional green tea leaves (n=61)

|  | Acetamiprid | Clothianidin | Dinotefuran | dm-acetamiprid | Imidacloprid | Nitenpyram | Thiacloprid | Thiamethoxam |
| --- | --- | --- | --- | --- | --- | --- | --- | --- |
| Acetamiprid | 1.000 |  |  |  |  |  |  |  |
| Clothianidin | -0.080 | 1.000 |  |  |  |  |  |  |
| Dinotefuran | 0.078 | 0.293* | 1.000 |  |  |  |  |  |
| dm-acetamiprid | 0.041 | 0.100 | -0.096 | 1.000 |  |  |  |  |
| Imidacloprid | 0.094 | 0.189 | 0.325* | 0.151 | 1.000 |  |  |  |
| Nitenpyram | 0.841** | -0.091 | 0.011 | 0.085 | -0.022 | 1.000 |  |  |
| Thiacloprid | 0.197 | 0.204 | 0.356* | -0.020 | 0.341* | 0.248 | 1.000 |  |
| Thiamethoxam | -0.016 | 0.383* | 0.451** | 0.093 | 0.354* | 0.057 | 0.413** | 1.000 |

Spearman Rho`s nonparametric correlation test; ***** *p* > 0.05, ***p* > 0.001

**Table S3*:*** Maximum Residual Limits (MRLs) (mg/kg) of neonicotinoids in tea leaves.

| Neonicotinoid | Japan | USA | EU | CODEX |
| --- | --- | --- | --- | --- |
| Clothianidin | 50 | 70* | 0.7 | 0.7 |
| Dinotefuran | 25 | - | - | - |
| Thiamethoxam | 20 | 20** | 20 | 20 |
| Thiacloprid | 30 | - | 10 | - |
| Nitenpyram | 10 | - | - | - |

*No US regulations [1].

**There are no US regulations as of March 27, 2013 [1].

**Table S4*:*** Physicochemical characteristics of the target neonicotinoid compounds [2,3].

| Neonicotinoid | Water Solubility (mg/L) | Vapor Pressure (Pa) | Log K_ow_ | Soil Half-Life (days) |
| --- | --- | --- | --- | --- |
| Acetamiprid | 4,250 (25°C) | 1 × 10−6 (25°C) | 0.8 | 3 (4–7) |
| Clothianidin | 327 (20°C) | 1.3 × 10−10 (25°C) | 0.91 | 545 (13–1,386) |
| Imidacloprid | 610 (20°C) | 4 × 10−10 (25°C) | 0.57 | 191 (104–228) |
| Nitenpyram | 600,000 (20°C) | <1 × 10−4 (50°C) | -0.64 | - |
| Thiacloprid | 185 (20°C) | 8 × 10−10 (25°C) | 1.26 | 15.5 (9–27) |
| Thiamethoxam | 4,100 (25°C) | 2.7 × 10−9 (20°C) | −0.13 | 50 (7–72) |
|  |  | 6.6 × 10−9 (25°C) |  |  |
| Dinotefuran | 39,830 (25°C) | <1.7 × 10−6 (30°C) | −0.55 | 82 |

**Table S5:** Criteria for Japanese Agricultural Standard (JAS) for organic agricultural product and its associated legislations [4]

| **JAS criteria/Legislations** | **Guidelines/Provisions** |
| --- | --- |
| Criteria for the method of production provided by article 2 | Natural recycling function of agriculture should be maintained or increased by:   - nonuse of chemically synthesized fertilizers and agricultural chemicals. - exercising the productivity of the soil; and - applying the cultivation method to minimize load to the environment as much as possible |
| Criteria for the method of production provided by article 4 | **Conditions for fields**   - Use of composts and nonuse of prohibited agricultural chemicals and fertilizers for not less than 2 years before sowing and planting (3 years for perennial plants) - Keeping field under proper management so as to prevent drifting and flowing of prohibited substances during the production periods.   **Manuring practice**   - The productivity of the soil should be maintained or increased by composts from residues of products in the fields, and the use of functions of organism in the fields or in the surrounding areas. - The use of fertilizers and soil conditioners may be permitted only in cases where the methods identified above are not effective for maintaining or increasing the productivity of the soil. |
| Criteria for seeds and seedlings | - Use of organically produced seeds and seedlings. - Use of seeds for seed reproductive plants and the youngest available seedlings for vegetative reproductive plants. - Nonuse of recombinant DNA technology |
| Relevant legislations | - The Law Concerning Standardization and Proper Labeling of Agricultural and Forestry Products (JAS Law) (Law of No. 175, 1950) - Enforcement Ordinance of the JAS Law - Enforcement Regulation of the JAS Law - Technical criteria for certifying production process managers etc. of organic agricultural products and organic feeds, etc. - The inspection method of production process of organic agricultural products, organic processed foods, organic feeds and organic livestock products |

**Table S6:** Concentrations of NEOs in green tea leaves (ng/g) compared with urinary concentrations of NEOs in human populations (ng/mL)

| Sample/country |  | ACE | dm-ACE | CLO | DIN | IMI | NIT | TCP | THXM | *ΣNEO* | LOQ |
| --- | --- | --- | --- | --- | --- | --- | --- | --- | --- | --- | --- |
| **Tea leaves/** | ***%Df*** | ***4.9*** | ***63.1*** | ***69.9*** | ***74.8*** | ***69.9*** | ***1.9*** | ***84.5*** | ***58.3*** | ***-*** |  |
| **Current study** | **median** | **BDL** | **0.36** | **2.02** | **8.3** | **1.45** | **BDL** | **0.3** | **0.63** | **17.63** | **0.05-1.33** |
|  | **Maximum** | **14.41** | **1.27** | **328.59** | **3407.24** | **14.16** | **0.08** | **11.99** | **959.4** | **3449.09** |  |
| Urine/Japan [5] | *%Df* | *56.0* | *-* | *96.0* | *100.0* | *96.0* | *29.0* | *67.0* | *100.0* | *-* |  |
| (N=52) | median | 0.02 | - | 0.7 | 2.3 | 1.9 | ND | 0.14 | 0.5 | 5.56 | 0.05-0.3 |
|  | Maximum | 0.36 | - | 6.6 | 27.4 | 8.2 | 1.03 | 0.5 | 6.2 | 49.26 |  |
| Urine/Japan [6] | *%Df* | *37.3* | *14.1* | *32.7* | *45.8* | *40.4* |  | *21.8* | *41.1* |  |  |
| (N=1036) | median | <LOQ | <LOQ | <LOQ | <LOQ | <LOQ | - | <LOQ | <LOQ | - | 0.01-0.74 |
|  | Maximum | 8.3 | 53.3 | 94.5 | 217 | 21.5 | - | 0.6 | 222 | 617.2 |  |
| Urine/Japan [7] | *%Df* | *12.1* | *-* | *8.1* | *57.8* | *15.2* | *20.6* | *0.0* | *25.1* | *-* |  |
| *(N=703)* | median | <LOQ | - | <LOQ | 0.44 | <LOQ | <LOQ | <LOQ | <LOQ | 0.44 | 0.03-1.07 |
|  | Maximum | 2.01 | - | 8.62 | 62.25 | 2.52 | 0.98 | <LOQ | 3.71 | 80.09 |  |
| Urine/Ghana [2] | *%Df* | *2.7* | *94.7* | *40.0* | *12.0* | *70.7* | *18.7* | *2.7* | *18.7* | *-* |  |
| (N=75) | Median | <LOQ | 0.41 | <LOQ | <LOQ | 0.15 | <LOQ | <LOQ | <LOQ | 1.1 | 0.05-0.2 |
|  | Maximum | 0.08 | 8.79 | 0.45 | 1.01 | 165.01 | 0.14 | 0.14 | 0.21 | 233.19 |  |
| Urine/China [8] | *%Df* | *96.0* | *-* | *99.0* | *96.0* | *97.0* | *-* | *92.0* | *98.0* | *-* |  |
| (N=324) | Median | 0.01 | - | 0.24 | 0.14 | 0.21 | - | <LOQ | 0.15 | 0.75 | 0.0007- |
|  | Maximum | 0.61 | - | 17 | 18 | 7.3 | - | <LOQ | 8.4 | 51.31 | 0.002 |
| Urine/China [9] | *%Df* | *38.0* | *96.0* | *96.6* | *69.0* | *86.0* | *-* | *-* | *90.7* | *-* |  |
| (N=129) | Median | <LOQ | 0.75 | 0.29 | 0.16 | 0.08 | - | - | 0.21 | 1.49 | 0.01-0.05 |
|  | Maximum | 0.93 | 18.3 | 15.8 | 7.8 | 0.86 | - | - | 11.7 | 55.39 |  |
| Urine/USA [10] | *%Df* | *20.0* | *25.0* | *85.0* | *-* | *70.0* | *-* |  | *55.0* | *-* |  |
| (N=20) | Median | <LOQ | <LOQ | 0.22 | - | 0.07 | - | <LOQ | 0.08 | 0.37 | 0.01-0.05 |
|  | Maximum | 0.25 | O.32 | 5.72 | - | - | - | <LOQ | 0.81 | 6.78 |  |

ACE: Acetamiprid, dm-ACE: N-desmethyl-acetamiprid, CLO: clothianidin, DIN: dinotefuran, IMI: imidacloprid, NIT: nitenpyram, TCP: thiacloprid, THXM: thiamethoxam, LOQ: limit of quantification, *Σ*NEO: total neonicotinoid concentration, %DF: percentage detection frequency.

1. Y. Ikenaka, Y. Miyabara, T. Ichise, S. M. M. Nakayama, C. Nimako, M. Ishizuka, C. Tohyama, Exposures of children to neonicotinoids in pine wilt disease control areas. Environ. *Toxicol. Chem.* 38(2019),71-79. doi: 10.1002/etc.4316.
2. C. Nimako, Y. Ikenaka, O. Akoto, N. Bortey-Sam, T. Ichise, S.M.M. Nakayama, A.K. Asante, K. Fujioka, K. Taira, M. Ishizuka, Human Exposures to Neonicotinoids in Kumasi, Ghana. *Environ Toxicol Chem.* (2021) 1-13, https://doi.org/10.1002/etc.5065.
3. R. Raina-Fulton, Neonicotinoid Insecticides: Environmental Occurrence in Soil, Water and Atmospheric Particles. Pesticides, (2016) chapter 2, 2-38. Available from: https://www.researchgate.net/profile/Renata_Raina-Fulton_bailey/publication/309174307_Neonicotinoid_Insecticides_Environmental_Occurrence_in_Soil_Water_and_Atmospheric_Particles/links/580248e008ae6c2449f7f937.pdf.
4. Ministry of Agriculture, Forestry and Fisheries. Organic JAS (2007). Available online: https://www.maff.go.jp/e/policies/standard/specific/organic_JAS.html. (accessed on 08 January 2021).
5. J. Ueyama, H.K. Harada, A. Koizumi, Y. Sugiura, T. Kondo, I. Saito, M. Kamijima, Temporal level of urinary neonicotinoid and dialkylphosphate concentrations in Japanese women between 1994 and 2011. *Environ. Sci. Technol.* 49(201, 14522–14528.
6. A. Osaka, J. Ueyama, T. Kondo, H. Nomura, Y. Sugiura, I. Saito, K. Nakane, A. Takaishi, H. Ogi, S. Wakusawa, Y. Ito, M. Kamijima, Exposure characterization of three major insecticide lines in urine of young children inJapan-neonicotinoids, organophosphates, and pyrethroids. *Environ. Res.,* 147(2016), 89−96.
7. N. Oya, Y. Ito, T. Ebara, S. Kato, J. Ueyama, A. Aoi, K. Nomasa, H. Sato, T. Matsuki, M. Sugiura-Ogasawara, S. Saitoh, M. Kamijima, Cumulative exposure assessment of neonicotinoids and investigation into their intake-related factors in young children in Japan. *Sci. total Environment*, 750(2021), 141630. https://doi.org/10.1016/j.scitotenv.2020.141630.
8. T. Zhang, S. Song, X. Bai, Y. He, B. Zhang, M. Gui, K. Kannan, S. Lu, Y. Huang, H. Sun, A nationwide survey of urinary concentrations of neonicotinoid insecticides in China. *Environ. Int.* 132(2019), 105114. https://doi.org/10.1016/j.envint. 2019.105114.
9. A. Wang, G. Mahai, Y. Wan, Z. Yang, Z. He, S. Xu, W. Xia, Assessment of imidacloprid related exposure using imidacloprid-oleﬁn and desnitro-imidacloprid: Neonicotinoid insecticides in human urine in Wuhan, China. *Environ. Int.* 141(2020), 105785. https://doi.org/10.1016/j.envint.2020.105785.
10. M. Honda, R. Robinson, K. Kannan, A simple method for the analysis of neonicotinoids and their metabolites in human urine, *Environ. Chem.* (2019). https://doi.org/10.1071/EN18240.
